# Association between dietary inflammatory index and osteoporosis in the US population: evidence from NHANES 2003–2010

**DOI:** 10.3389/fnut.2025.1508127

**Published:** 2025-01-29

**Authors:** Zhiwen Liu, Huanling Jian, Zijing Peng, Sicheng Xiong, Zhihai Zhang

**Affiliations:** ^1^The Third Clinical College of Guangzhou University of Chinese Medicine, Guangzhou, China; ^2^The Third Affiliated Hospital of Guangzhou University of Chinese Medicine, Guangzhou, China

**Keywords:** NHANES, DII, osteoporosis, cross-sectional study, LASSO

## Abstract

**Objective:**

This study aimed to explore the association between the Dietary Inflammatory Index (DII) and the prevalence of osteoporosis in the U.S. population, using data from the National Health and Nutrition Examination Survey (NHANES) 2003–2010.

**Methods:**

Data from 7,290 participants in the NHANES 2003–2010 survey were analyzed. The relationship between the DII and osteoporosis was evaluated using weighted multivariate logistic regression, and potential non-linear associations were explored through restricted cubic spline (RCS) regression. Subgroup analyses were conducted with stratified models, and the findings were depicted in a forest plot. To pinpoint key dietary factors associated with osteoporosis, we applied least absolute shrinkage and selection operator (LASSO) regression. These factors were integrated into a nomogram for risk prediction, with the model’s discriminative ability assessed via the receiver operating characteristic (ROC) curve.

**Results:**

Osteoporosis patients had higher DII scores than those without the condition (1.61 vs. 1.18, *p* < 0.001). After adjusting for covariates, participants in the highest DII quartile had an 88% greater risk of osteoporosis (OR: 1.88, 95% CI: 1.41–2.52, *P* for trend <0.001). Restricted cubic spline analysis confirmed a linear relationship between DII and osteoporosis risk. Subgroup analyses showed similar patterns across different groups, as illustrated by the forest plot. LASSO regression identified key dietary factors, which were used to build a nomogram with an AUC of 83.6%, indicating strong predictive accuracy.

**Conclusion:**

A higher DII is strongly linked to increased osteoporosis risk, underscoring the importance of reducing dietary inflammation to help prevent osteoporosis.

## Introduction

Osteoporosis, a progressive condition characterized by low bone mineral density (BMD) and compromised bone structure, is a leading cause of fractures, particularly among postmenopausal women and the elderly (1, 2). With the global population aging, the prevalence of osteoporosis is expected to rise, placing a heavy burden on healthcare systems due to the increased risk of fractures, particularly hip and vertebral fractures. These fractures are often associated with reduced mobility, higher mortality, and significant healthcare costs, underscoring the importance of early detection and prevention (3, 4). Inflammation plays a key role in bone metabolism and has been implicated in the pathogenesis of osteoporosis (5). Chronic, low-grade inflammation accelerates bone resorption by osteoclasts and impairs bone formation by osteoblasts, leading to a net loss of bone mass. Pro-inflammatory cytokines such as tumor necrosis factor-alpha (TNF-*α*) and interleukin-6 (IL-6) have been shown to promote bone resorption, while anti-inflammatory strategies can help protect bone health (6).

Dietary patterns can significantly influence levels of systemic inflammation. The Dietary Inflammatory Index (DII) is a tool designed to quantify the inflammatory potential of an individual’s diet based on their intake of various pro-and anti-inflammatory nutrients (7). A higher DII score reflects a diet that promotes inflammation, while a lower DII score indicates an anti-inflammatory diet (8). Prior research has established links between higher DII scores and an increased risk of chronic conditions like cardiovascular disease, diabetes, and some cancers (9). Despite this, few studies have directly investigated the relationship between dietary inflammation and osteoporosis risk. Given the well-established role of inflammation in bone loss, it is plausible that diets high in pro-inflammatory foods may contribute to the development of osteoporosis. However, this area remains underexplored, particularly in large, diverse populations.

This study aims to fill this gap by examining the association between the Dietary Inflammatory Index and osteoporosis using data from the National Health and Nutrition Examination Survey (NHANES) 2003–2010. By investigating this relationship, we aim to better understand whether diets with higher inflammatory potential are linked to a higher risk of osteoporosis. Furthermore, we will use advanced statistical techniques, including LASSO regression, to identify key dietary factors that contribute to osteoporosis risk and develop a predictive nomogram for individualized risk assessment.

The NHANES dataset was selected for its representativeness of the US population and its comprehensive dietary and health data. DII was chosen as it quantitatively evaluates the inflammatory potential of diet based on established evidence.

## Methods

### Participants in NHANES

**Figure 1:**
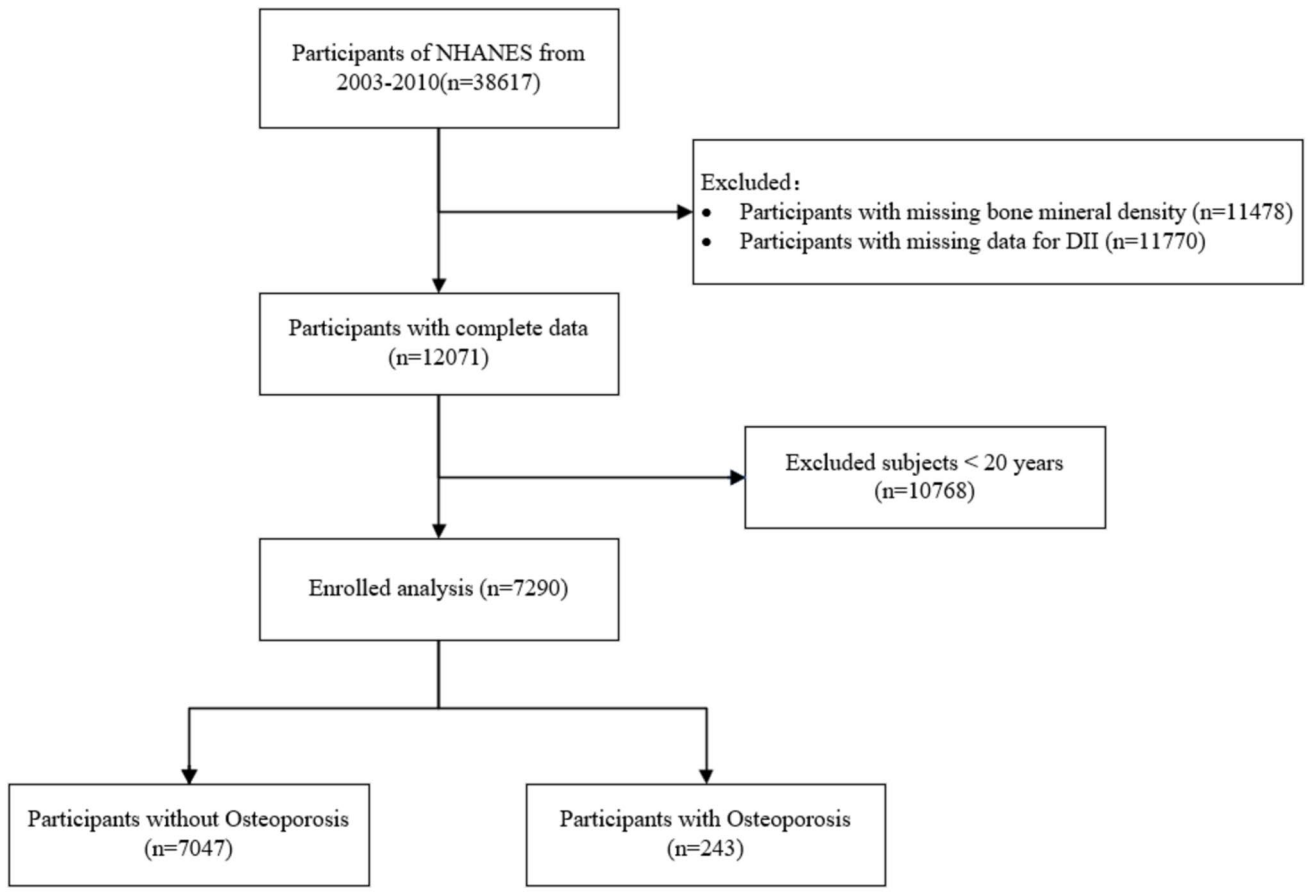
Flowchart of sample selection.

This study used data from the NHANES from 2003 to 2010. Initially, 38,617 participants were considered. We excluded 11,478 individuals due to missing BMD data and 11,770 participants for missing DII data. After these exclusions, 12,071 participants remained with complete data. Additionally, participants under the age of 20 (*n* = 10,768) were excluded, leaving a final sample of 7,290 adults. These individuals were divided into two groups: 7,047 participants without osteoporosis and 243 participants diagnosed with osteoporosis. Figure 1 illustrates the specific procedure.

### The exposure and outcome variables definition

The key exposure variable in this study was the DII. DII is a literature-based tool used to assess the inflammatory potential of an individual’s diet (10, 11). It ranks dietary intake on a scale from anti-inflammatory to pro-inflammatory, based on the effects of various nutrients on inflammatory biomarkers. In this study, due to limitations in the NHANES dataset, only 27 of the original 45 food parameters were used, including macronutrients like carbohydrates, protein, fats, fiber, and micronutrients such as vitamins A, B, C, E, iron, magnesium, and others. These dietary components were collected through 24-h dietary recall interviews. While this partial list may impact the comprehensiveness of DII scoring, these components cover the most relevant dietary contributors to inflammation. DII scores were calculated by standardizing nutrient intakes (*Z*-scores), adjusting them according to their inflammatory effects, and summing them to create a total score for each participant. For analysis, participants were divided into quartiles, from the lowest (most anti-inflammatory) to the highest (most pro-inflammatory) (12).

The primary outcome of this study was osteoporosis. Referring to previous studies, dual-energy X-ray (DXA) scans of the spine (L1–L4) or both hip joints were performed to evaluate an individual’s BMD (QDR 4500A fan-beam densitometers [Hologic Inc.]). Osteoporosis was defined as a *T*-score  ≤  − 2.5 at either the femoral neck or the lumbar spine. *T*-scores were calculated as (mean BMD respondent group—mean BMD reference group)/SD reference group. The reference group for calculation of the femoral neck consisted of 20–29 white females from the NHANES III report.

### Covariates in NHANES

The covariates were selected from those previously reported factors to influence osteoporosis. Age, race, gender, and body mass index (BMI) are examples of demographic characteristics. The socio-economic covariates include education level, marital status. Covariates on health-related behaviors: smoking and alcohol consumption. Variables related to medical comorbidities: uric acid, phosphorus, serum calcium, Alanine Aminotransferase (ALT), Aspartate Aminotransferase (AST), alkaline phosphatase (ALP), blood urea nitrogen (BUN), hypertension, and hyperlipidemia. Missing data in covariates were filled by interpolation.

### Statistical analysis

All statistical analyses were performed using R software (version 4.3.2), incorporating NHANES survey weights to account for the complex sampling design. Descriptive statistics were calculated, with continuous variables presented as means and standard deviations (SD) if the data were normally distributed, and as medians with interquartile ranges (IQR) for non-normally distributed variables. Normality of the data distribution was tested using the Shapiro–Wilk test. For normally distributed variables, comparisons were performed using *t*-tests, while for non-normally distributed variables, Mann–Whitney *U* tests were applied. Categorical variables were reported as frequencies and percentages, with comparisons made using chi-square tests.

To assess the association between the DII and osteoporosis, weighted multivariate logistic regression models were applied. Three models were constructed to progressively adjust for potential confounders. Model 1 included basic adjustments for age, gender, and race. Model 2 expanded these adjustments by incorporating socioeconomic factors such as education level, marital status, and family income-to-poverty ratio. Model 3 was the fully adjusted model, further accounting for lifestyle factors (smoking status, BMI) and clinical measures (serum calcium, ALT, AST, uric acid, alkaline phosphatase, blood urea nitrogen, phosphorus, hypertension, and hyperlipidemia).

Additionally, restricted cubic spline (RCS) regression was used to examine any potential non-linear relationships between DII and osteoporosis risk. Subgroup analyses were conducted to evaluate the consistency of the DII-osteoporosis relationship across various population subgroups, and the results were displayed in a forest plot. LASSO regression was applied to identify key dietary factors influencing osteoporosis risk, which were then incorporated into a risk prediction nomogram. The performance of the nomogram was evaluated using the receiver operating characteristic (ROC) curve, with the area under the curve (AUC) used to assess predictive accuracy. A *p*-value of <0.05 was considered statistically significant.

## Results

### Baseline characteristics of participants

Table 1 summarizes the baseline characteristics of the 7,290 participants, categorized into osteoporosis (*n* = 243) and non-osteoporosis (*n* = 7,047) groups. The mean age of participants with osteoporosis was significantly higher (70.6 years) compared to those without osteoporosis (49.9 years, *p* < 0.001). Osteoporosis was more prevalent among females (75.7%) than males (24.3%, *p* < 0.001). Racial disparities were observed, with a higher proportion of white individuals in the osteoporosis group (73.7%) and lower proportions of Black (8.23%) and Mexican (11.5%) individuals (*p* < 0.001). Additionally, osteoporosis patients had a significantly lower mean body mass index (BMI) of 24.6 compared to 28.2 in non-osteoporosis individuals (*p* < 0.001). Other notable differences included higher prevalence of hypertension and hyperlipidemia among participants with osteoporosis (*p* < 0.001 and *p* = 0.014, respectively).

**Table 1:** Baseline characteristics of the study participants grouped by osteoporosis status.

	Total	No-osteoporosis	Osteoporosis	*P*-value
	*N* = 7,290	*N* = 7,047	*N* = 243	
Age (years)	50.6 (18.8)	49.9 (18.6)	70.6 (12.9)	<0.001***
Gender				<0.001***
Female	3,499 (48.0%)	3,315 (47.0%)	184 (75.7%)	
Male	3,791 (52.0%)	3,732 (53.0%)	59 (24.3%)	
Race				<0.001***
Black	1,465 (20.1%)	1,445 (20.5%)	20 (8.23%)	
Mexican	1,415 (19.4%)	1,387 (19.7%)	28 (11.5%)	
Other	496 (6.80%)	480 (6.81%)	16 (6.58%)	
White	3,914 (53.7%)	3,735 (53.0%)	179 (73.7%)	
Education level				0.373
Above high school	3,500 (48.0%)	3,394 (48.2%)	106 (43.6%)	
High school or equivalent	1825 (25.0%)	1760 (25.0%)	65 (26.7%)	
Under high school	1965 (27.0%)	1893 (26.9%)	72 (29.6%)	
Marital status				<0.001***
Living alone	2,577 (35.3%)	2,455 (34.8%)	122 (50.2%)	
Married/Living with partner	4,713 (64.7%)	4,592 (65.2%)	121 (49.8%)	
Ratio of family income to poverty level				0.006**
<1.0	1,265 (17.4%)	1,223 (17.4%)	42 (17.3%)	
≥3.0	3,031 (41.6%)	2,952 (41.9%)	79 (32.5%)	
1.0 ~ 2.9	2,994 (41.1%)	2,872 (40.8%)	122 (50.2%)	
BMI	28.1 (5.83)	28.2 (5.84)	24.6 (4.37)	<0.001***
Calcium (mg/dL)	9.53 (0.36)	9.53 (0.35)	9.53 (0.38)	0.879
ALT (U/L)	25.6 (28.0)	25.7 (28.4)	20.8 (14.3)	<0.001***
AST (U/L)	25.9 (23.5)	25.9 (23.8)	25.2 (8.42)	0.269
Uric acid (U/L)	5.43 (1.35)	5.44 (1.35)	5.09 (1.33)	<0.001***
ALP (IU/L)	70.9 (28.0)	70.5 (23.3)	84.5 (87.2)	0.013*
BUN (mg/dl)	13.0 (5.69)	12.9 (5.55)	15.6 (8.41)	<0.001**
Phosphorus (mg/dl)	3.80 (0.53)	3.79 (0.53)	3.91 (0.61)	0.003**
Smoking				0.761
No	3,622 (49.7%)	3,502 (49.8%)	118 (48.6%)	
Yes	3,663 (50.2%)	3,538 (50.2%)	125 (51.4%)	
Hypertension				<0.001***
No	4,177 (57.3%)	4,080 (57.9%)	97 (39.9%)	
Yes	3,113 (42.7%)	2,967 (42.1%)	146 (60.1%)	
Hyperlipidemia				0.014*
No	6,181 (84.8%)	5,989 (85.0%)	192 (79.0%)	
Yes	1,109 (15.2%)	1,058 (15.0%)	51 (21.0%)	

Mean ± SD for continuous variables: the p value was calculated by the weighted linear regression model. (%) for categorical variables: the *P*-value was calculated by the weighted chi-square test. *P-value < 0.05, **p-value < 0.01, ***p-value < 0.001.

Table 2 presents dietary data and the calculated Dietary Inflammatory Index (DII) across the same groups. The DII score was significantly higher in participants with osteoporosis (mean 1.61) compared to those without osteoporosis (mean 1.18, *p* < 0.001). Participants with osteoporosis had lower intake of energy (1,618 vs. 2,164 kcal, *p* < 0.001), protein, carbohydrates, and fats, all showing significant differences. For example, the average protein intake in the osteoporosis group was 61.2 g, compared to 82.0 g in the non-osteoporosis group (*p* < 0.001). Nutrient intakes such as magnesium, iron, and zinc were also significantly lower in the osteoporosis group.

**Table 2:** Dietary intake of each DII component grouped by osteoporosis status.

	Total	No-osteoporosis	Osteoporosis	*P*-value
	*N* = 7,290	*N* = 7,047	*N* = 243	
DII	1.20 (1.82)	1.18 (1.82)	1.61 (1.71)	<0.001***
Energy	2,146 (1013)	2,164 (1018)	1618 (638)	<0.001***
Protein	81.3 (42.4)	82.0 (42.7)	61.2 (28.0)	<0.001***
Carbohydrate	261 (131)	262 (132)	208 (83.9)	<0.001**
Dietary fiber	15.5 (9.40)	15.6 (9.45)	14.1 (7.58)	0.003**
Total fat	80.8 (46.0)	81.5 (46.2)	60.5 (34.5)	<0.001***
Total saturated fat	26.6 (16.7)	26.8 (16.7)	20.8 (13.1)	<0.001***
MUFA	30.2 (18.1)	30.5 (18.2)	21.8 (12.9)	<0.001***
PUFA	17.1 (11.4)	17.3 (11.5)	12.8 (9.04)	<0.001***
Cholesterol	297 (241)	300 (242)	210 (178)	<0.001***
Vitamin A	594 (750)	593 (758)	616 (479)	0.466
β carotene	1911 (3526)	1898 (3520)	2285 (3698)	0.110
Vitamin B1	1.61 (0.94)	1.63 (0.95)	1.32 (0.57)	<0.001***
Vitamin B2	2.17 (1.24)	2.18 (1.25)	1.90 (0.86)	<0.001***
Niacin	24.0 (13.8)	24.2 (13.9)	18.4 (8.66)	<0.001***
Vitamin B6	1.89 (1.20)	1.90 (1.21)	1.54 (0.80)	<0.001***
Total, folate	392 (234)	394 (235)	334 (170)	<0.001***
Vitamin B12	0.83 (2.11)	0.83 (2.12)	0.90 (1.75)	0.557
Vitamin C	90.1 (105)	90.4 (106)	80.5 (71.0)	0.037*
Vitamin E	0.42 (2.47)	0.42 (2.48)	0.33 (1.99)	0.511
Magnesium	281 (145)	282 (146)	248 (100)	<0.001***
Iron	15.5 (8.84)	15.6 (8.90)	13.2 (6.63)	<0.001***
Zinc	11.9 (9.74)	12.0 (9.84)	9.23 (5.79)	<0.001***
Selenium	108 (61.2)	109 (61.6)	81.6 (38.7)	<0.001***
Caffeine	167 (216)	167 (216)	167 (226)	0.992
Alcohol	11.2 (28.8)	11.5 (29.2)	3.31 (10.6)	<0.001***

Data of dietary intake of DII components are presented as weighted mean [95% CI]. DII, dietary inflammatory index; MUFA, monounsaturated fatty acid; PUFA, polyunsaturated fatty acid. *P-value < 0.05, **P-value < 0.01, ***P-value < 0.001.

### Association of DII with osteoporosis

The relationship between the DII and osteoporosis was assessed using multivariate logistic regression. After adjusting for demographic and clinical variables, participants in the highest DII quartile (Q4) had a significantly higher risk of osteoporosis compared to those in the lowest quartile (Q1). In the fully adjusted model (Table 3), the odds ratio (OR) for Q4 was 1.88 (95% CI: 1.41–2.52, *P* for trend <0.001), indicating a strong association between pro-inflammatory diets and increased osteoporosis risk.

**Table 3:** Odds ratios for associations between DII and osteoporosis.

Model	Quartiles of DII				*P* for trend
	Q1	Q2	Q3	Q4	
Model 1	1.00	1.55 (1.17–2.07)	1.89 (1.43–2.28)	2.24 (1.72–2.95)	<0.001***
Model 2	1.00	1.46 (1.09–1.95)	1.71 (1.29–2.27)	1.92 (1.46–2.55)	<0.001***
Model 3	1.00	1.50 (1.12–2.03)	1.67 (1.25–2.24)	1.88 (1.41–2.52)	<0.001***

Model 1 was adjusted for age, sex, race.

Model 2 was adjusted for age, sex, race, education level, marital status, ratio of family income to poverty level.

Model 3 was adjusted for age, race, sex, education level, marital status, ratio of family income to poverty level, smoking, BMI, Calcium, ALT, AST, uric acid, ALP, BUN, phosphorus, hypertension, hyperlipidemia. *P-value < 0.05, **p-value < 0.01, ***p-value < 0.001.

**Figure 2:**
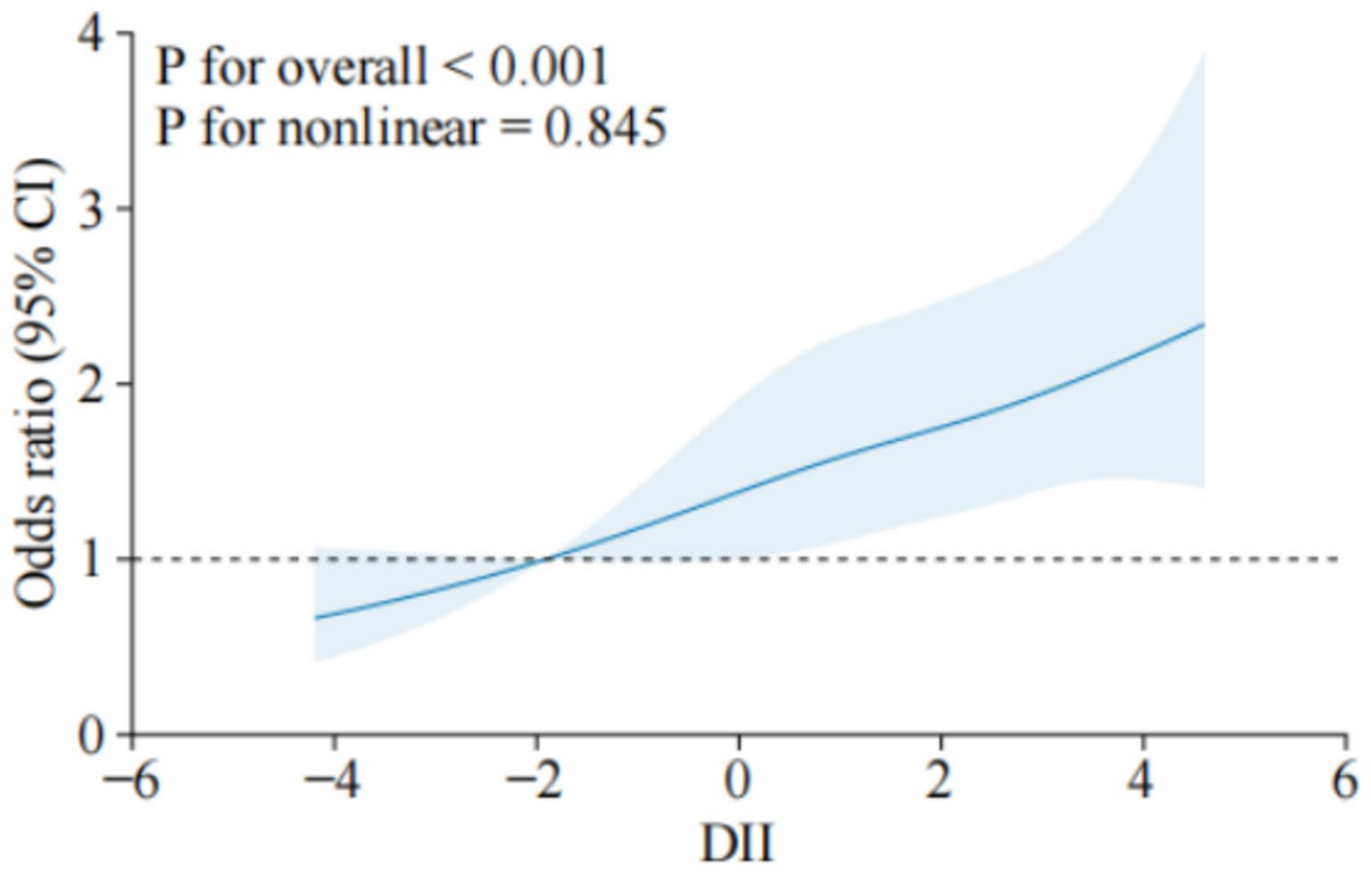
Dose–response relationship between DII and osteoporosis. The solid line indicates the estimated risk of osteoporosis, and the dashed line indicates the fitted 95% CI.

The RCS analysis further confirmed a linear increase in osteoporosis risk as DII scores rose. The overall association was significant (*p* < 0.001), and no evidence of non-linearity was found (*p* = 0.845), as shown in Figure 2. This suggests that as dietary inflammation increases, the risk of osteoporosis rises proportionally.

**Figure 3:**
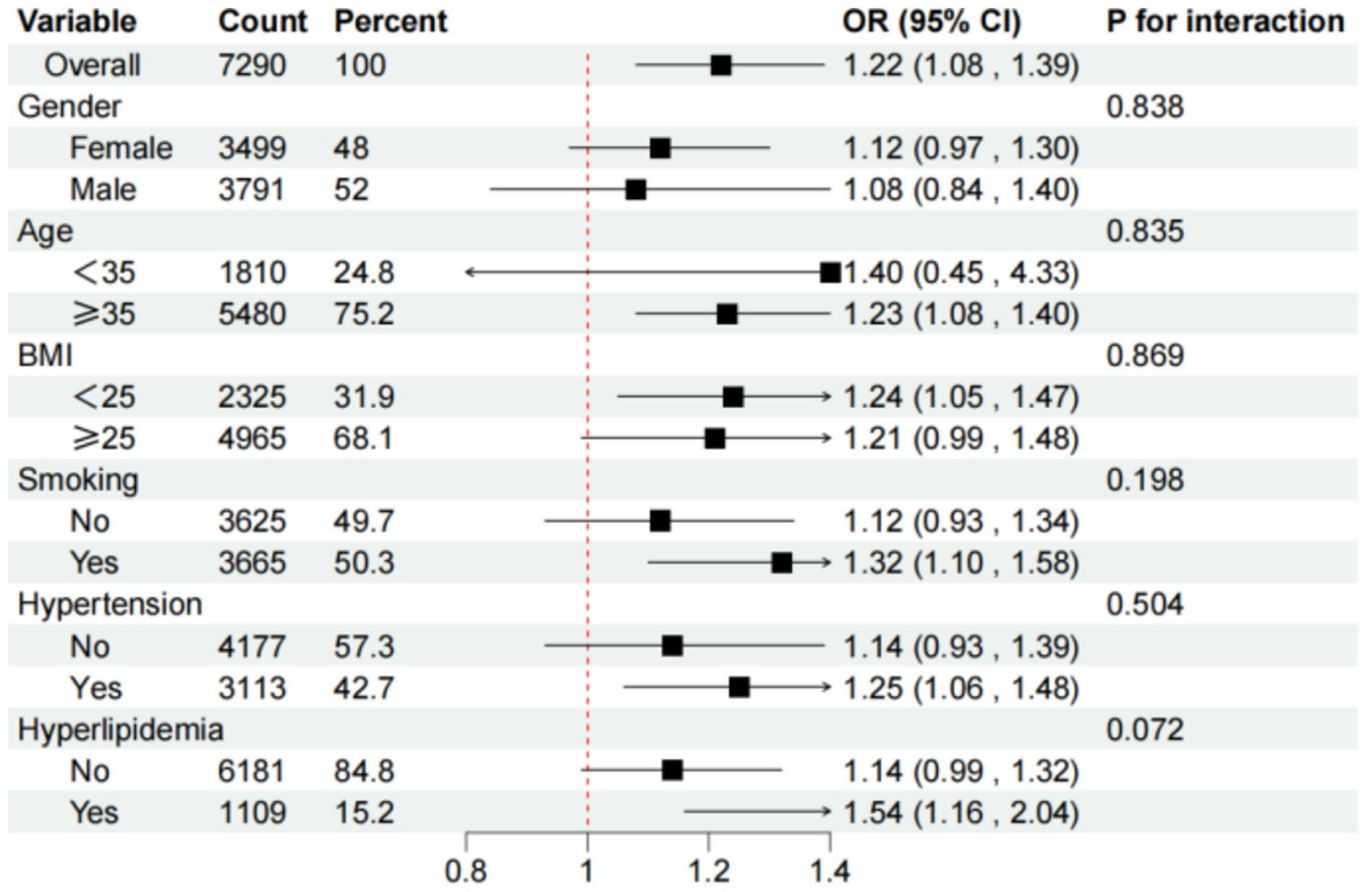
Subgroups analyses for the association between DII and osteoporosis.

Subgroup analyses, visualized in Figure 3, demonstrated that the positive association between higher DII scores and osteoporosis remained consistent across different demographic and clinical subgroups. These findings underscore dietary inflammation as a significant risk factor for osteoporosis, independent of factors such as age, gender, or comorbidities.

### Identification of key osteoporosis-related dietary factors

**Figure 4:**
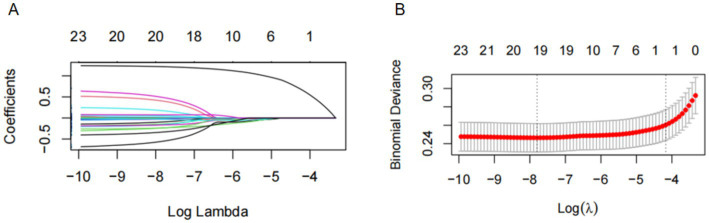
The LASSO penalized regression analysis for identifying key osteoporosis-related dietary factors. **(A)** The coefficient shrinkage process of all 25 dietary components and 1 covariate (age), we represent the changes in coefficients of different features under various levels of shrinkage by drawing lines of different colors. **(B)** A 10-fold cross-validation of the LASSO regression model. LASSO, least absolute shrinkage and selection operator.

The LASSO penalized regression was applied to identify the dietary factors most closely associated with osteoporosis risk. This method helps refine the model by shrinking the coefficients of less relevant variables to zero, leaving only the most important predictors. In this analysis, 25 dietary components were evaluated, alongside key covariates such as age, sex, race/ethnicity, body mass index (BMI), smoking status, and several clinical indicators, including serum calcium, ALT, AST, uric acid, alkaline phosphatase (ALP), blood urea nitrogen (BUN), and phosphorus levels (Figure 4A). To ensure optimal variable selection, 10-fold cross-validation was performed to find the best penalty (lambda) that minimized binomial deviance. This process resulted in the selection of key dietary factors, including carbohydrates, dietary fiber, cholesterol, polyunsaturated fatty acids (PUFA 22:6 n-3), iron, and alcohol. These dietary elements were found to be most strongly linked to osteoporosis risk, based on the LASSO feature selection process (Figure 4B).

**Figure 5:**
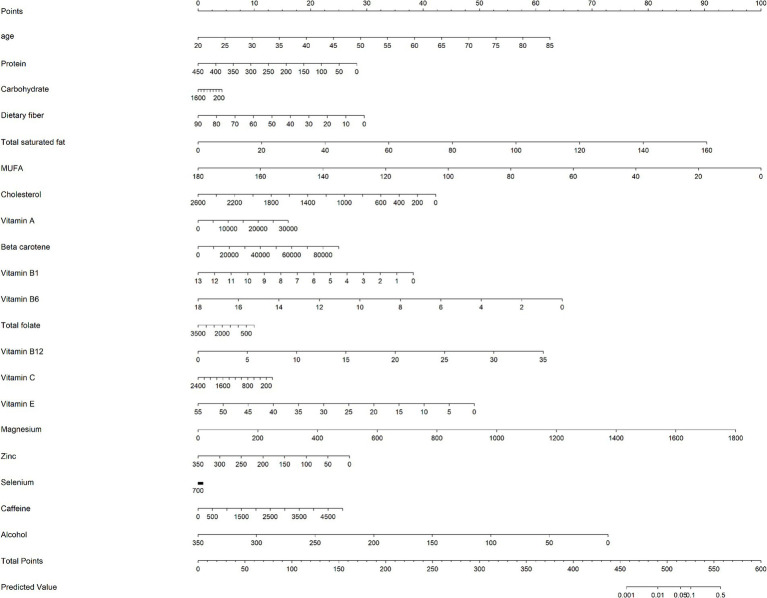
A nomogram model based on age, and 6 key osteoporosis-related dietary factors identified by LASSO regression analysis.

**Figure 6:**
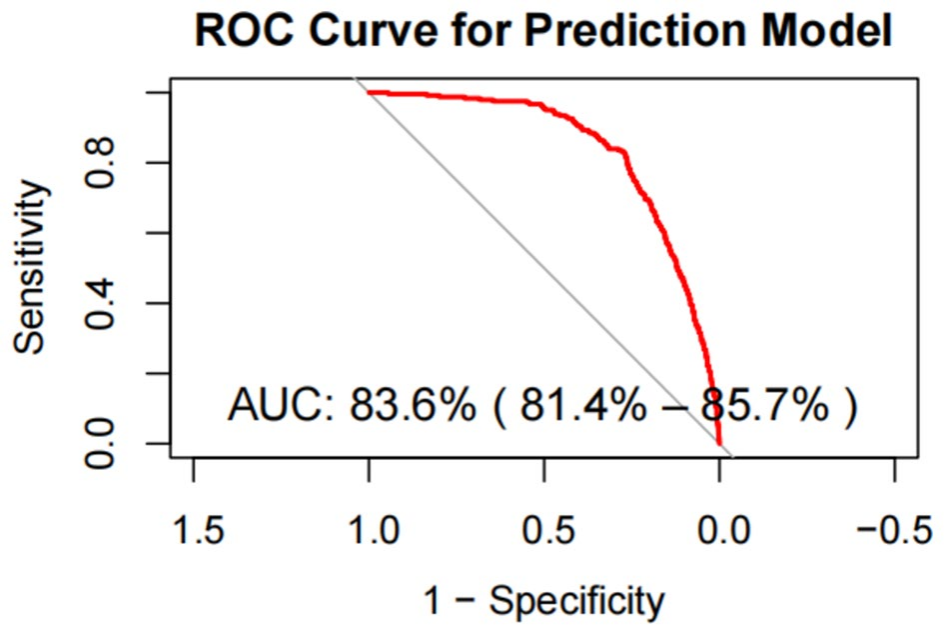
ROC curve for evaluating the predictive power for osteoporosis of the nomogram model.

Using these key dietary factors and covariates, a nomogram was constructed to predict individualized osteoporosis risk. The nomogram integrates both dietary and clinical factors, allowing for a personalized risk assessment (Figure 5). This tool highlights how specific dietary components can impact bone health and provides a practical framework for targeting preventive interventions for individuals at higher risk of osteoporosis. The performance of the risk prediction model was validated through the ROC curve analysis, yielding an AUC of 83.6%, indicating strong discriminatory power in distinguishing between individuals with and without osteoporosis (Figure 6). This demonstrates that the combination of dietary factors and clinical data provides a reliable tool for assessing osteoporosis risk.

## Discussion

This study explored the association between the DII and osteoporosis in a representative sample of the U.S. population using NHANES data from 2003 to 2010. Our findings revealed a strong, positive association between higher DII scores and increased risk of osteoporosis. Participants in the highest DII quartile had a significantly higher risk of osteoporosis, with an 88% increased risk compared to those in the lowest quartile. These findings are consistent with prior research indicating that pro-inflammatory diets contribute to lower BMD and greater fracture risk. Inflammatory cytokines, such as IL-6 and TNF-*α*, are known to mediate bone resorption by promoting osteoclast activity while inhibiting osteoblast function. This may explain the observed association between higher DII scores and increased osteoporosis risk.

It is important to note that this study utilized a subset of 27 dietary parameters from the original 45 parameters in the DII calculation, as limited by the NHANES dataset. Previous studies, such as Xu et al. (13) incorporated the full set of 45 parameters, potentially allowing for a more comprehensive assessment of dietary inflammatory potential. However, the parameters included in this study represent the most critical contributors to dietary inflammation and bone health, as established in prior research. While the reduction in parameters may slightly influence the DII scoring range and its comparability with other studies, it is unlikely to significantly alter the observed association with osteoporosis risk. Future studies incorporating the full set of DII parameters could help validate these findings and further standardize DII-based analyses.

Several studies have reported similar associations between the DII and bone health outcomes. For example, a systematic review by Veronese et al. found that higher DII scores were associated with a significantly increased risk of fractures in women, particularly those postmenopausal, over an eight-year follow-up (14, 15). Similarly, a longitudinal study by Cervo et al. (16) demonstrated that a higher dietary inflammatory load was linked to reduced BMD and greater fracture risk in older adults (17). These results align with our findings, suggesting that inflammatory diets may exacerbate bone loss through heightened osteoclast activity and reduced osteoblast function, both of which are mediated by inflammatory cytokines. Mazidi et al. also reported a significant inverse relationship between DII and BMD in U.S. adults, supporting the hypothesis that pro-inflammatory diets contribute to bone deterioration (18, 19). Another study conducted by Orchard et al. (20) on postmenopausal women showed that those with higher DII scores were more likely to experience hip and vertebral fractures, further emphasizing the negative impact of dietary inflammation on bone health (21). In our study, key dietary components such as carbohydrates, dietary fiber, cholesterol, and PUFAs (particularly 22:6 *n*-3) were identified as significant contributors to osteoporosis risk. These findings are supported by existing research. PUFAs, especially omega-3 fatty acids, have been shown to possess anti-inflammatory properties that promote bone health (22, 23). Conversely, diets high in refined carbohydrates and cholesterol may increase systemic inflammation, leading to an elevated risk of osteoporosis. Studies have consistently shown that individuals with higher DII scores have elevated levels of these inflammatory biomarkers, which are linked to poorer bone health outcomes. For instance, Shivappa et al. (12) found a significant correlation between higher DII scores and increased CRP levels, suggesting a direct link between dietary inflammation and systemic inflammation. The pro-inflammatory effects of certain foods—such as those high in saturated fats, refined sugars, and cholesterol—have been associated with reduced BMD and greater risk of osteoporosis. Given the aging global population and the rising prevalence of osteoporosis, these findings have important public health implications. Dietary interventions that reduce the consumption of pro-inflammatory foods may provide a cost-effective strategy to prevent osteoporosis. Previous research has suggested that adherence to anti-inflammatory diets—such as the Mediterranean diet, which is rich in fruits, vegetables, whole grains, and healthy fats—can significantly reduce inflammation and improve bone health (24, 25). A meta-analysis by Fabiani et al. (26) found that individuals who adhered to anti-inflammatory diets had significantly lower levels of inflammatory markers and better BMD outcomes. Similarly, research by Zheng et al. (27) on Chinese adults showed that those who consumed diets rich in anti-inflammatory foods had a lower risk of hip fractures. These studies suggest that incorporating anti-inflammatory dietary practices may be an effective means of preventing bone loss and reducing fracture risk, particularly in older populations.

Meanwhile, recent advancements in bone-targeted therapies provide innovative solutions for treating osteoporosis. For example, Cui et al. (28) developed engineered exosomes to deliver siRNA, effectively suppressing inflammation-related bone resorption. Similarly, Cui et al. (29) introduced bioinspired nanovesicles to modulate the skeletal microenvironment and restore bone homeostasis. Cui et al. (30) further demonstrated that biomimetic nanogels could re-establish osteoblast/osteoclast balance, offering targeted treatment for postmenopausal osteoporosis. These emerging therapies complement dietary strategies by directly targeting the inflammatory mechanisms underlying osteoporosis.

This study has several strengths, including the use of a large, nationally representative sample and robust statistical techniques such as LASSO regression and restricted cubic spline analysis. These methods allowed us to isolate the most significant dietary predictors of osteoporosis and examine the dose–response relationship between DII and osteoporosis risk. However, the study also has limitations. The cross-sectional design precludes establishing causality, and dietary intake data were self-reported, which may introduce recall bias. Additionally, although we adjusted for multiple potential confounders, residual confounding from unmeasured factors cannot be entirely ruled out. This study has inherent limitations, including its cross-sectional design, which precludes causal inferences. Additionally, the reliance on self-reported dietary data may introduce recall bias, and residual confounding from unmeasured variables such as physical activity cannot be excluded.

Future research should focus on longitudinal studies to establish causal relationships between dietary inflammation and bone health. Furthermore, personalized nutrition approaches, which consider genetic and environmental factors, may provide deeper insights into the individual variability in responses to dietary interventions. Given the significant role of diet in modulating inflammation, such strategies could be particularly useful in preventing osteoporosis and improving bone health outcomes in at-risk populations.

## Conclusion

In conclusion, this study demonstrates a significant association between higher DII scores and increased osteoporosis risk. Pro-inflammatory diets, characterized by specific nutrients like refined carbohydrates and cholesterol, are linked to bone loss, while anti-inflammatory components such as polyunsaturated fatty acids (PUFAs) show protective effects. These findings emphasize the importance of promoting anti-inflammatory dietary patterns as part of osteoporosis prevention strategies, particularly in older adults. Future research should focus on longitudinal studies to further validate these associations and explore personalized dietary interventions to reduce osteoporosis risk.

## Data Availability

The datasets presented in this study can be found in online repositories. The names of the repository/repositories and accession number(s) can be found in the article/supplementary material.
